# Supplementary material for: The Microbial Community Structure in the Rhizosphere of Theobroma cacao L. and Euterpe oleracea Mart. Is Influenced by Agriculture System in the Brazilian Amazon
Source: Microorganisms. 2024 Feb 17;12(2):398. doi: 10.3390/microorganisms12020398 (PMC10892126; doi:10.3390/microorganisms12020398)
Supplement: Supplementary file 1 [file microorganisms-12-00398-s001.zip › microorganisms-2802296-supplementary.pdf]

Supplementary Materials

Table S1. Quantitative test of antimicrobial substance (AMS) production by strain affiliated with the genus *Bacillus* sp.

**Bacterial strains**	**Inhibition (%)**					
	Colletotrichum	*Curvularia*	*Fusarium*	*Pestalotiopsis*	*Pythium*	*Rhizoctonia*
CP1-28	59,15±3,00a	71,76±2,52a	75,71±2,00ab	47,15±1,15a	62,50±1,00ab	68,66±1,73ab
CP1-30	59,62±1,52a	76,85±1,15ab	84,29±9,54ab	56,91±1,15a	50,00±7,55a	65,17±1,15a
CP2-47	63,38±1,00a	77,78±2,65ab	85,24±9,07ab	58,54±1,00a	55,09±2,31ab	68,66±1,00ab
CP2-38	60,56±0,00a	75,00±4,36ab	66,19±3,06a	64,23±6,81a	60,65±2,31ab	72,14±2,52ab
CP2-13	62,91±1,52a	75,93±0,58ab	73,81±1,15ab	63,41±3,61a	61,57±7,23ab	69,65±3,79ab
CP3-40	100,00±0,00c	100,00±0,00c	100,00±0,00b	100,00±0,00b	69,44±0,00b	100,00±0,00c

Means ± SD with common letters in the same column do not show significant differences (p<0,05) by Tukey's test.

A

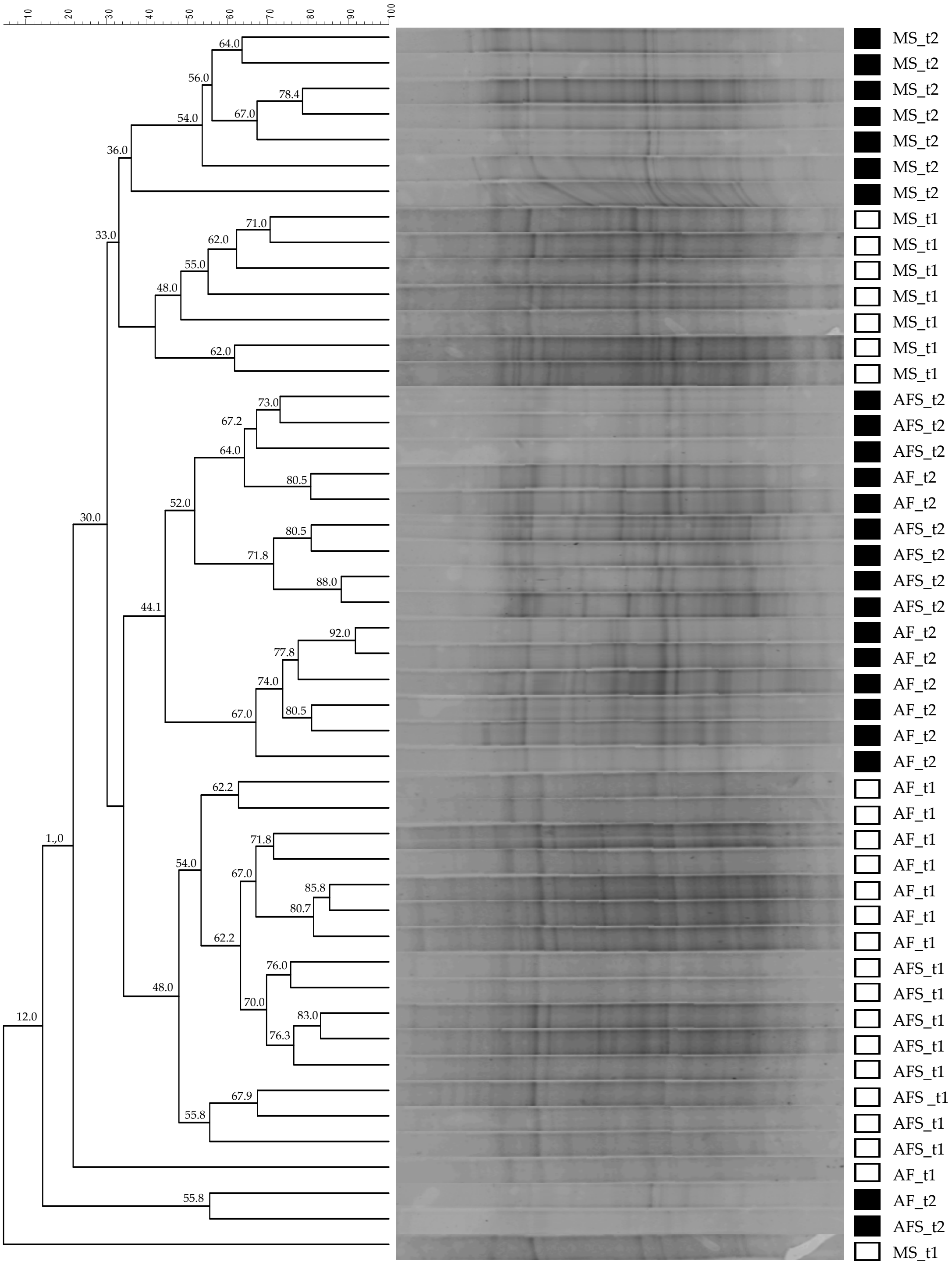
10
20
30
40
50
60
70
80
90
100
64.0
56.0
78.4
67.0
54.0
36.0
33.0
71.0
62.0
55.0
48.0
62.0
73.0
67.2
64.0
80.5
52.0
30.0
80.5
71.8
88.0
44.1
92.0
77.8
74.0
80.5
67.0
62.2
1.,0
71.8
54.0
67.0
85.8
80.7
62.2
76.0
48.0
70.0
83.0
12.0
76.3
67.9
55.8
55.8
MS_t2
MS_t2
MS_t2
MS_t2
MS_t2
MS_t2
MS_t2
MS_t1
MS_t1
MS_t1
MS_t1
MS_t1
MS_t1
MS_t1
AFS_t2
AFS_t2
AFS_t2
AF_t2
AF_t2
AFS_t2
AFS_t2
AFS_t2
AFS_t2
AF_t2
AF_t2
AF_t2
AF_t2
AF_t2
AF_t2
AF_t1
AF_t1
AF_t1
AF_t1
AF_t1
AF_t1
AF_t1
AFS_t1
AFS_t1
AFS_t1
AFS_t1
AFS_t1
AFS _t1
AFS_t1
AFS_t1
AF_t1
AF_t2
AFS_t2
MS_t1

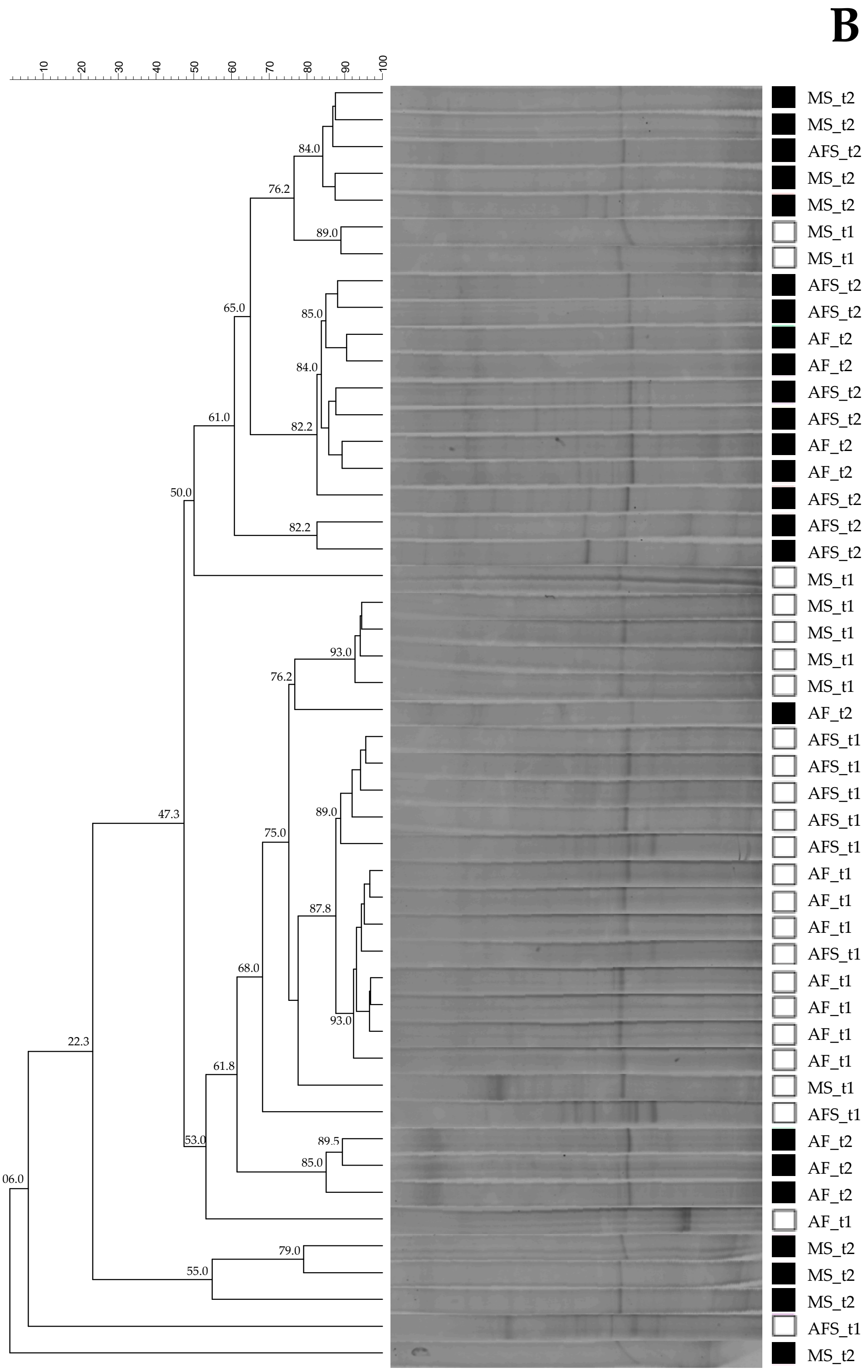
B
10 20 30 40 50 60 70 80 90 100
84.0
76.2
89.0
65.0
85.0
84.0
61.0
82.2
50.0
82.2
93.0
76.2
47.3
89.0
75.0
87.8
68.0
93.0
22.3
61.8
89.5
53.0
85.0
06.0
79.0
55.0
MS_t2
MS_t2
AFS_t2
MS_t2
MS_t2
MS_t1
MS_t1
AFS_t2
AFS_t2
AF_t2
AF_t2
AFS_t2
AFS_t2
AF_t2
AF_t2
AFS_t2
AFS_t2
AFS_t2
MS_t1
MS_t1
MS_t1
MS_t1
MS_t1
AF_t2
AFS_t1
AFS_t1
AFS_t1
AFS_t1
AFS_t1
AF_t1
AF_t1
AF_t1
AFS_t1
AF_t1
AF_t1
AF_t1
AF_t1
MS_t1
AFS_t1
AF_t2
AF_t2
AF_t2
AF_t1
MS_t2
MS_t2
MS_t2
AFS_t1
MS_t2

C

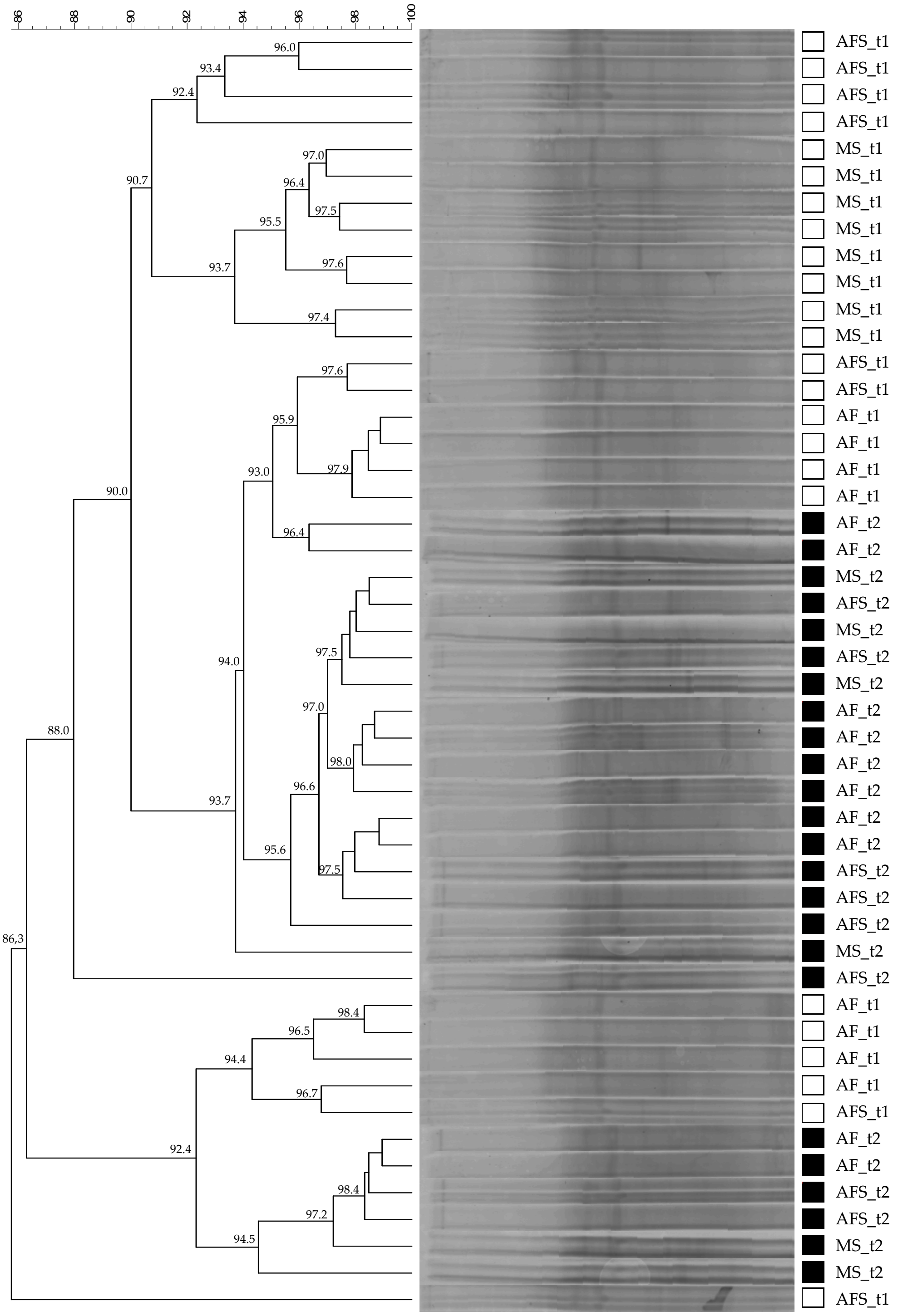

86
88
90
92
94
96
98
100
96.0
93.4
92.4
90.7
97.0
96.4
97.5
95.5
97.6
93.7
97.4
97.6
95.9
93.0
97.9
90.0
96.4
94.0
97.5
97.0
88.0
98.0
96.6
93.7
95.6
97.5
86,3
98.4
96.5
94.4
96.7
92.4
98.4
97.2
94.5
AFS_t1
AFS_t1
AFS_t1
AFS_t1
MS_t1
MS_t1
MS_t1
MS_t1
MS_t1
MS_t1
MS_t1
MS_t1
AFS_t1
AFS_t1
AF_t1
AF_t1
AF_t1
AF_t1
AF_t2
AF_t2
MS_t2
AFS_t2
MS_t2
AFS_t2
MS_t2
AF_t2
AF_t2
AF_t2
AF_t2
AF_t2
AF_t2
AFS_t2
AFS_t2
AFS_t2
MS_t2
AFS_t2
AF_t1
AF_t1
AF_t1
AF_t1
AFS_t1
AF_t2
AF_t2
AFS_t2
AFS_t2
MS_t2
MS_t2
AFS_t1

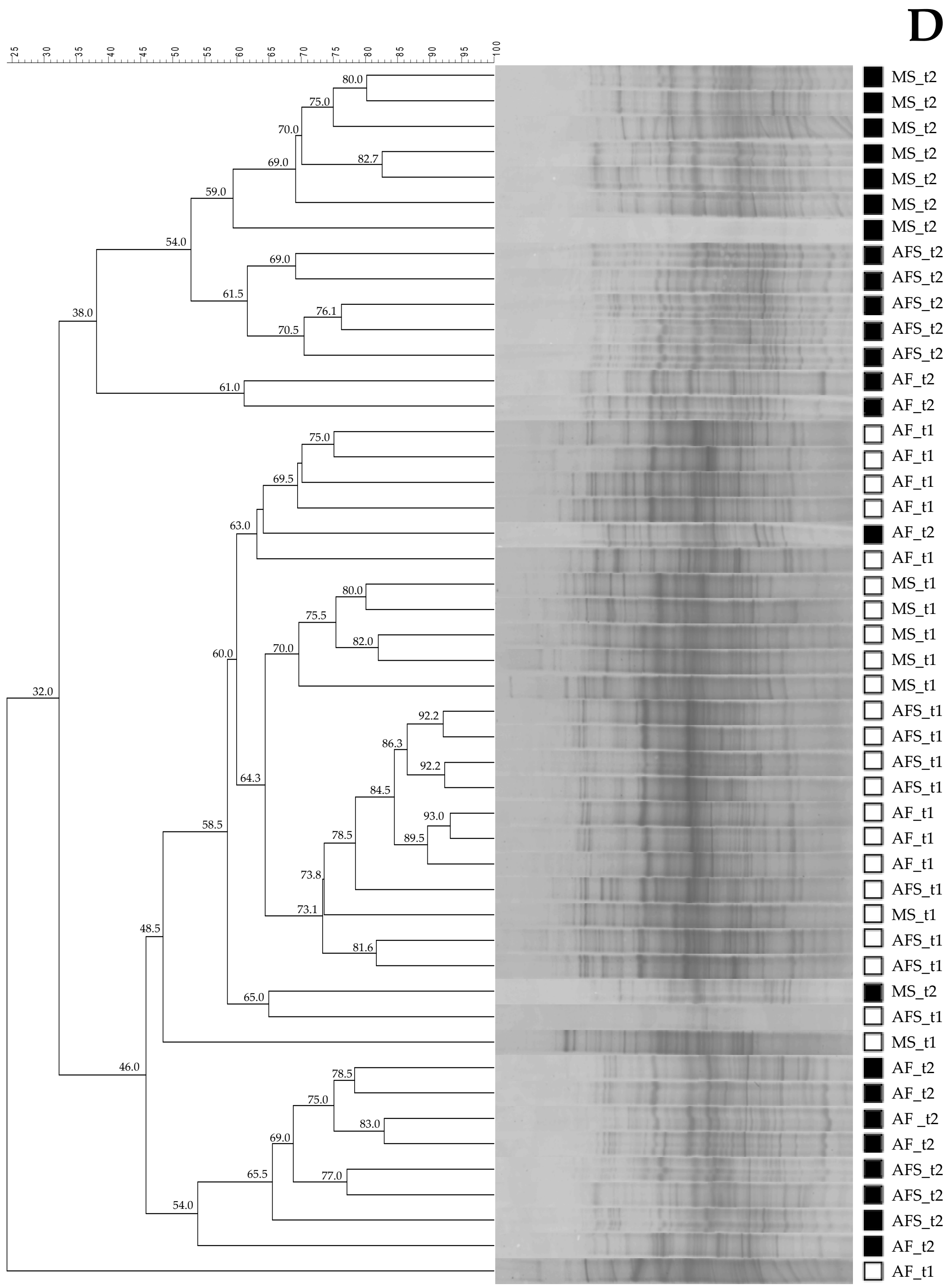

Figure S1. UPGMA cluster analysis of the DGGE fingerprint of bacterial communities (A), phosphate-solubilizing bacteria (B), diazotrophic (C), and fungi (D) in AFS, MS e AF systems during dry (t1) and rainy (t2) seasons.
